# Visceral Obesity and Metabolic Dysfunction in IgA Nephropathy: Nutritional and Metabolic Perspectives on Disease Progression

**DOI:** 10.3390/nu17203307

**Published:** 2025-10-21

**Authors:** Agnieszka Skibicka, Sylwia Małgorzewicz

**Affiliations:** 1 Medical Department, CSL Behring, 17 Branickiego Street, 02-972 Warsaw, Poland; 2 Department of Clinical Nutrition, Medical University of Gdańsk, Skłodowskiej-Curie 3a, 80-210 Gdańsk, Poland; sylwia.malgorzewicz@gumed.edu.pl; 3 Department of Nephrology, Transplantology and Internal Diseases, UCC Gdańsk, 80-952 Gdańsk, Poland

**Keywords:** IgA nephropathy, visceral obesity, metabolic syndrome, nutrition, dietary interventions, progression of chronic kidney disease

## Abstract

Introduction: IgA nephropathy (IgAN) is the most common primary glomerulonephritis in the world. In addition to genetic and immunological factors, visceral obesity and metabolic syndrome (MetS) are the main determinants of disease progression. This review aims to critically assess the role of visceral obesity and metabolic syndrome in driving the progression of IgA nephropathy (IgAN), with an emphasis on their underlying pathophysiological mechanisms and clinical implications. Methods: A systematic review was carried out in accordance with PRISMA guidelines. PubMed was searched (2015–2025) using terms related to IgA nephropathy, obesity, metabolic syndrome, and immunometabolic pathways. Only English-language observational and clinical studies in adults, excluding pediatric and animal studies, were included in the review. Additional sources were consulted to give context to the mechanistic aspects of obesity-related IgAN progression. Results: Visceral obesity and MetS accelerate IgAN progression through endocrine, inflammatory, and immune pathways, including cytokines derived from visceral adipose tissue, adipokines, intestinal dysbiosis, and BAFF/APRIL-mediated immune activation. MetS patients had higher proteinuria, a faster decrease in eGFR, and a higher risk of end-stage renal failure (23/65 vs. 15/60 endpoints, *p* < 0.001). Nutritional and metabolic interventions—including weight reduction, GLP-1 receptor agonists, dual GLP-1/GIP agonists, and bariatric/metabolic surgery—demonstrate renoprotective effects in obesity-related kidney disease and may have implications for IgAN. Conclusions: Obesity should be considered a chronic disease and a modifiable risk factor for IgAN. Nutrition-focused interventions targeting visceral obesity and metabolic dysfunction can slow the progression of the disease and should be included in renal guidelines. This review expands current knowledge by demonstrating that when sequential steps of IgAN pathophysiology are mapped with respect to endocrine and immunological effects of visceral adipose tissue, they converge on the same proinflammatory and immune pathways. This convergence suggests a bidirectional amplification loop in which obesity accelerates IgAN progression and increases the burden of complications.

## 1. Introduction

IgA nephropathy (IgAN) is the most common primary glomerulonephritis in the world and is a significant clinical problem due to its chronic, progressive nature. The disease is characterized by the deposition of immune complexes containing immunoglobulin A1 in the glomerular mesangia, which leads to chronic kidney damage and interstitial fibrosis [1,2,3]. The pathogenesis of IgAN is multifactorial and includes dysregulation of the immune system, abnormal glycosylation of IgA1 molecules, as well as genetic and environmental factors [3,4,5].

IgA nephropathy is more common in young adults, and its clinical presentation is heterogeneous—ranging from asymptomatic microscopic hematuria to rapidly progressive glomerulonephritis. The natural course of the disease is highly variable and often unpredictable. While some patients may maintain stable renal function for years, others experience a gradual decline in kidney function, typically reflected by a persistent reduction in estimated glomerular filtration rate (eGFR) or sustained proteinuria exceeding 1 g/day. Persistent albuminuria (≥300 mg/day) and hypertension are recognized markers of disease progression and inflammatory/repair biomarkers have been implicated in risk stratification [1,6,7,8]. In a significant proportion of patients, IgA nephropathy ultimately leads to chronic kidney disease and, in advanced stages, end-stage renal failure requiring renal replacement therapy such as dialysis or kidney transplantation [1,8].

In recent years, more and more attention has been paid to the influence of metabolic factors on the progression of IgA nephropathy. Obesity, insulin resistance, and metabolic syndrome are associated with increased proteinuria and accelerated loss of renal function in patients with IgAN [2,9,10,11,12,13]. These mechanisms include chronic low-grade inflammation, oxidative stress, and activation of the renin–angiotensin–aldosterone system, all of which exacerbate glomerular damage and promote fibrosis [9,14,15,16]. Clinical studies have shown that the presence of obesity and components of metabolic syndrome are independent risk factors for poor prognosis in IgAN, highlighting the need for a comprehensive therapeutic approach that includes not only blood pressure control and immunosuppression, but also lifestyle modification and treatment of concomitant metabolic disorders [10,11,12,13,17,18,19,20,21,22,23].

Due to its high prevalence and serious clinical implications, IgA nephropathy remains the subject of intensive research, focusing both on the identification of prognostic biomarkers and on the search for new therapeutic strategies [8,11,24,25].

It is now known that in the pathophysiology of IgA nephropathy (IgAN), the cytokines BAFF and APRIL play a key role in supporting B cell survival, increasing Gd-IgA1 synthesis, and promoting immune complex deposition in the mesangia, thereby accelerating damage to the glomerular [26] similar to obesity [27,28,29]. Given the increasing prevalence of overweight and obesity, also among people with glomerulonephritis, new concepts have been proposed, such as metabolic dysfunction-associated kidney disease (MDAKD) [30] and chronic metabolic kidney syndrome (CRMS). These units describe the mutual reinforcement of metabolic abnormalities and kidney dysfunction. A growing body of evidence suggests that the coexistence of IgAN with obesity and metabolic syndrome may represent an overlap syndrome in which metabolic-immune interactions significantly impair the prognosis [22,31].

Recently, MDAKD has been proposed as a new entity, defined as kidney disease occurring in the context of metabolic dysfunction (e.g., insulin resistance, dyslipidemia, central obesity, hypertension). In contrast to the traditional classification of CKD, which is mainly based on a decrease in eGFR and albuminuria, MDAKD emphasizes metabolic factors that cause kidney damage [30]. Similarly, the concept of CRMS describes a broader syndrome in which metabolic abnormalities and kidney dysfunction reinforce each other, creating a vicious circle that accelerates cardiovascular risk and progression of CKD [32,33,34,35] In this context, obesity, metabolic dysfunction, and IgAN may converge to form an overlap syndrome, where metabolic–immune interactions amplify disease activity beyond what would be anticipated from each condition individually [16,31].

However, the evidence for the effect of obesity on IgAN remains inconsistent when analyses are based solely on body mass index (BMI), which does not include the inflammatory VAT burden [13,17]. Stronger associations with IgAN progression have been noted when obesity coexists with hypertension, dyslipidemia, glomerular enlargement or changes in the gut microbiome [5,19,21,22].

This review aims to critically assess the role of visceral obesity and metabolic syndrome in driving the progression of IgA nephropathy (IgAN), with an emphasis on their underlying pathophysiological mechanisms and clinical implications.

## 2. Methods

The systematic review was carried out in accordance with the PRISMA guidelines [36]. A literature search between 1 January 2015 and 1 June 2025 and selected previous studies were conducted in PubMed using the keywords: “IgA nephropathy”, “obesity”, “visceral adipose tissue”, “metabolic syndrome”, “CRMS”, “MDAKD”, “chronic kidney disease”, “BAFF”, “APRIL”, “inflammation”, “adipokines”, “IgAN progression”, “complement system” and “intestinal dysbiosis”. Logical operators (AND, OR) were used in the searches, e.g., “IgA nephropathy AND obesity AND inflammation”. Filters included English-language articles, full-text articles, abstracts, clinical trials, and meta-analyses. The terms PubMed MeSH (e.g., “glomerulonephritis, IGA”, “Obesity”) were used.

After removing 353 duplicates from 1245 articles, 892 titles and abstracts were analyzed, excluding 720 articles. Additionally, we manually checked the literature lists to identify publications of interest.

The full texts of 172 publications were analyzed in terms of: (1) the effect of obesity, MetS or CRMS on the progression of IgAN; and (2) molecular mechanisms in the pathophysiology of obesity and IgAN. The variables analyzed included BMI, waist circumference, proteinuria, glomerular filtration rate (GFR), lipid profile, histological data, and cytokine levels (e.g., hsCRP). We focused on observational and clinical studies in adults, as recommended for systematic reviews. Studies in pediatrics populations and animal models were excluded and finally 54 papers were included for analysis.

Use of Artificial Intelligence Tools: Artificial intelligence (AI)–assisted tools, including ChatGPT (OpenAI) and Grok 4, were used during the preparation of this manuscript to support text editing, language refinement, and graphical presentation. All content was reviewed and verified by the authors for accuracy and scientific integrity.

**Figure 1:**
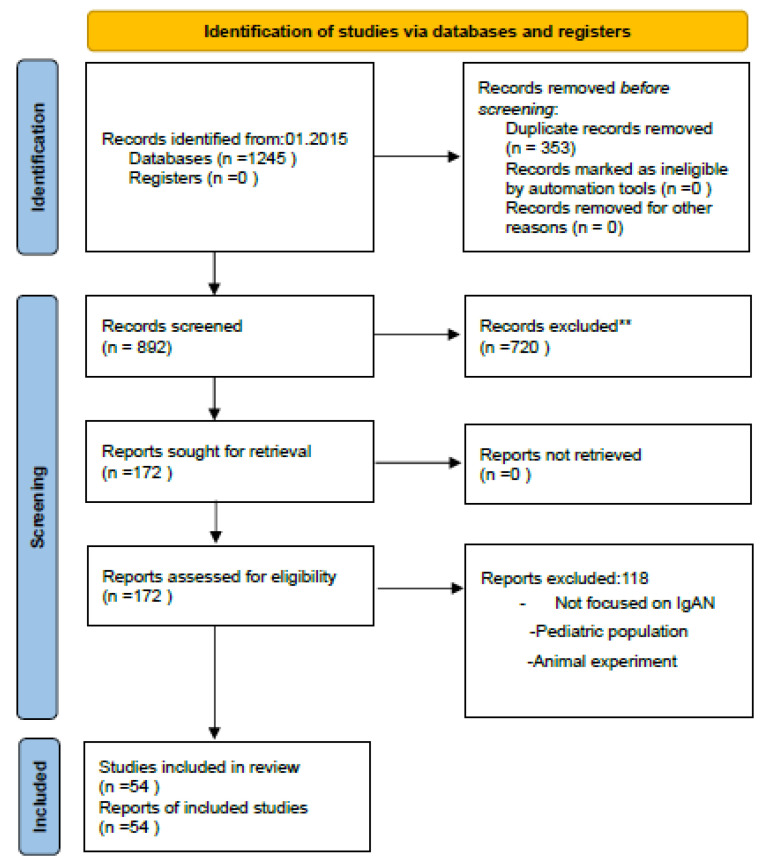
PRISMA 2020 Test Selection Flow Diagram. ** Records excluded during screening due to irrelevance to the research question or insufficient data.

While the PRISMA flowchart (Figure 1) documents papers that met the inclusion criteria for quantitative and qualitative synthesis, additional references not included in the PRISMA diagram were selectively cited to explain key pathophysiological mechanisms, particularly those related to immunometabolic pathways, adipokine signaling, and complement activation. This approach follows established methodological guidelines that allow for the integration of mechanistic and guideline-based evidence to contextualize clinical outcomes.

## 3. Results

### 3.1. Effect of Obesity, MetS on IgAN Progression

The authors of almost all studies emphasize that obesity interacts with hypertension, hyperuricemia, hypertriglyceridemia and histopathological changes, worsening event-free survival and renal outcomes [17,31]. Several studies have shown a detrimental role of a high BMI in the progression of IgAN. A meta-analysis confirmed that obesity increases the risk of kidney side effects [31], while cohort studies have shown delayed remission of proteinuria [17], severity of interstitial fibrosis [19], and worse long-term prognosis [20,21]. For example, BMI ≥ 25 kg/m^2^ combined with histopathological abnormalities significantly delayed proteinuria remission [17]. A greater decrease in eGFR has also been documented in obese patients [31]. In general, obese patients with IgAN have higher proteinuria, lower eGFR, and a higher incidence of hypertension, hyperuricemia, and hypertriglyceridemia compared to those with a normal BMI [21].

The results of the studies showed a relationship between BMI and some pathologies in patients with IgAN shown in Table 1.

The other studies presented in Table 2 show synergistic effects of BMI and other parameters such as intestinal fibrosis, matrix expansion, GBN thickness, metabolic factors. The authors of these publications noted an additional negative impact of high BMI in patients with IgAN. Wu Wang et al. [19] described, for example, the risk of interstitial fibrosis in patients with IgAN by BMI category. A strong association was observed in obese patients, especially with a BMI of ≥28 kg/m^2^, with interstitial fibrosis.

Several studies show a link between MetS and IgAN [10,11,12,22,42]. A comparison of clinical parameters in patients with IgAN with and without MetS is presented in Table 3. Patients with IgAN and MetS showed low eGFR, higher proteinuria, a higher number of cases of hypertension, cardiovascular events, and a significantly higher risk of ESRD [10,17,21,30,43]. In addition, disease progression correlates with the number of MetS components, with a gradual decline in renal outcomes as more metabolic abnormalities coexist [10,43].

However, the association between BMI and IgAN progression is not entirely consistent: some cohorts reported no significant effect [25], and others identified U-shaped risks, where underweight also increased the incidence of ESRD [12].

**Figure 2:**
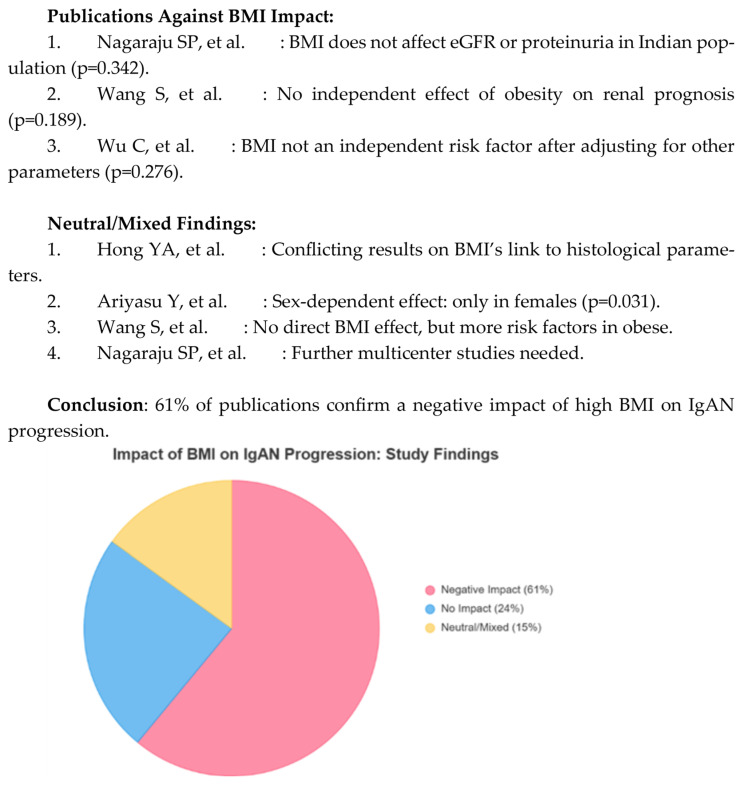
Effect of BMI on IgAN progression: study results [19,21,22,23,25].

In addition, some studies have found inconsistent associations between BMI and histopathological parameters. While higher BMI was associated with interstitial fibrosis, tubular atrophy, or mesangial proliferation in some cohorts, other analyses did not confirm these correlations. This variability likely reflects differences in study design, patient populations, and disease stage, highlighting the complexity of the relationship and the need for standardized histopathological assessments. Figure 2 shows publications indicating no association or conflicting results between BMI and IgA progression and those that did not.

Figure 2 summarizes 54 peer-reviewed publications, illustrating that approximately 61% support the negative effect of high BMI on IgAN progression, while 39% report no significant or mixed associations. This distribution highlights that while most of the evidence links visceral obesity and elevated BMI to worse renal outcomes in IgAN, a significant minority of studies require more contextual and multifactorial models integrating obesity with components of metabolic syndrome, fat distribution, and demographic factors.

From 54 peer-reviewed publications, about 61% of participants support the negative impact of a high BMI on IgAN progression, and 39% report no significant or mixed associations. This distribution highlights that while most of the evidence links visceral obesity and elevated BMI to worse renal outcomes in IgAN, a significant minority of studies require more contextual and multifactorial models integrating obesity with components of metabolic syndrome, fat distribution, and demographic factors.

### 3.2. Pathophysiological Mechanisms

VAT secretion of pro-inflammatory cytokines such as IL-6 and TNF-α, and adipokines, including leptin and chemerin [1,14,15], is also elevated in IgAN, suggesting that visceral obesity and IgAN share overlapping inflammatory pathways, potentially enhancing disease activity through an overlapping syndrome.

Another potential pathophysiological mechanism is intestinal dysbiosis, often seen in obese individuals, which increases the production of galactose-deficient IgA1 (Gd-IgA1) through the TLR4 signaling pathway, further driving immune activation [5]. In addition, elevated levels of BAFF and APRIL promote B cell survival and differentiation in obesity, linking metabolic dysfunction with increased autoimmunity and mesangial immune complex deposition [26,27,44]. In IgAN, BAFF and APRIL are now known to support B cell survival and differentiation for increased Gd-IgA1 synthesis, autoantibody production, and C3 complement deposition in the mesangium, thereby accelerating glomerular injury.

These findings indicate that VAT-induced inflammation, adipokine imbalance, BAFF/APRIL signaling, intestinal dysbiosis, and oxidative stress coincide with histopathological abnormalities to accelerate IgAN progression, moreover, circulating biomarkers of inflammation and tubular repair have been associated with CKD progression and may refine risk in IgAN [6]. While current evidence strongly suggests metabolic-immune synergies, inconsistencies between studies highlight the need for future studies using precise measures of obesity (e.g., VAT imaging, waist indicators) to determine the true impact of obesity on IgAN outcomes.

The characteristics of all included studies are summarized in Supplementary Table S1.

## 4. Discussion

This review highlights the important role of obesity and MetS in accelerating the progression of IgA nephropathy (IgAN). The results show that metabolic dysfunction is not a passive background factor, but an active factor causing the disease to worsen. Clinical evidence consistently shows that patients with IgAN and obesity or MetS have higher rates of renal and cardiovascular events, greater proteinuria, and a faster decrease in eGFR compared to those without obesity/MetS [10,11,12,13,17,18,19,20,21,22,23]. The cumulative load of multiple MetS components further increases the risk, indicating an “overlap syndrome” in which obesity and metabolic dysfunction interact synergistically with IgAN’s immune mechanisms [11,31]. These results strongly support the inclusion of nutritional and metabolic risk stratification in clinical practice [34,35].

At the molecular level, visceral adipose tissue acts as an endocrine organ, producing pro-inflammatory mediators (IL-6, TNF-α), adipokines (leptin, chemerin) and reducing adiponectin, thus favoring oxidative stress and damage to the glomeruli [14,16,45,46]. Obesity-associated intestinal dysbiosis disrupts the gut-kidney axis, activating GALT and increasing Gd-IgA1 production [5,47], while BAFF/APRIL overexpression links adipose tissue inflammation with increased B cell activation and complement deposition [8,26,27,43,44,48]. These overlapping pathways reinforce the hypothesis of a nutritional-immune interface in the pathogenesis of IgAN.

Importantly, therapeutic possibilities emerge from this integration of nephrology and nutritional sciences. Weight reduction through lifestyle interventions, medical nutritional therapy, and structured dietary counseling can alleviate the progression of IgAN [34,35,49,50]. In clinical practice, it is often difficult to untangle the individual influence of diet, physical activity, and lifestyle modifications on outcomes such as body weight, blood pressure, salt intake, and overall diet quality. These factors often overlap and bring cumulative benefits. Therefore, they should be considered as elements of a comprehensive lifestyle intervention in which the synergistic impact of the combined modifications is clinically more relevant than the isolated contribution of each individual component.

Pharmacological treatments such as GLP-1 receptor agonists and dual GLP-1/GIP agonists, extensively studied in metabolic diseases, offer hope for renoprotection [32]. Bariatric and metabolic surgery have shown sustained improvements in renal outcomes in obesity-related kidney disease, and their role in IgAN needs to be investigated [35,49,50].

### 4.1. Effect of Obesity and MetS on IgAN Progression

The analysis of the literature shows that high BMI and MetS significantly accelerate the progression of IgAN [10,11,12,13,17,18,19,20,21,22,23]. MetS increases the risk of kidney failure through elevated blood pressure, diabetes, and dyslipidemia, contributing to cardiovascular and renal events. A 2024 study in Biomedicines found that IgAN patients with MetS experienced significantly more endpoints (23/65 vs. 15/60, *p* < 0.001), with independent predictors such as dyslipidemia, eGFR, hemoglobin, albuminuria, and diabetes [11]. A higher number of MetS components was associated with an increased risk of shortened renal survival (*p* = 0.012) [11]. Some authors emphasize the high cardiovascular risk and propose the term metabolic cardiorenal syndrome integrating metabolic, cardiovascular and renal dysfunction, intensifying inflammation through complement activation and endothelial dysfunction [11]. In addition, the concept MDAKD emphasizes the role of visceral obesity and insulin resistance in kidney pathology [30].

### 4.2. Molecular Mechanisms in the Pathophysiology of Obesity and IgAN

Obesity exacerbates IgAN through multiple molecular pathways. VAT acts as an endocrine organ, secreting IL-6, TNF-α, leptin, and chemerin, increasing Gd-IgA1 production, autoantibody formation, and complement activation [5,14,44]. Low levels of adiponectin reduce anti-inflammatory protection, increasing oxidative stress [8,15].

Intestinal dysbiosis, with altered Firmicutes/Bacteroidetes ratios, disrupts the gut-renal axis, promoting the overproduction of Gd-IgA1 through TLR4 [5,45]. Recent research has suggested that obesity-related alterations in the gut microbiome may promote increased Gd-IgA1 [5]. Elevated levels of BAFF and APRIL further increase B cell activity, autoantibody production, and complement deposition [5,43,44]. BAFF also intensifies insulin resistance through NF-κB and JNK signaling [27,44].

Obesity promotes lipotoxicity, complement activation, mesangial proliferation and fibrosis [44,51]. A 2025 Nature Reviews nephrology review confirmed that IL-6 and TNF-α derived from VAT directly contribute to renal inflammation [51]. Endothelin-1 worsens vasoconstriction and podocyte damage [52]. Biomarkers such as hsCRP and YKL-40 correlate with higher proteinuria and lower eGFR [53] consistent with broader evidence that inflammatory and repair biomarkers track CKD progression [6]. These common pathways suggest an overlap syndrome in which obesity reinforces the four-hit model of IgAN. Although VAT-induced inflammation, adipokine imbalance, and BAFF/APRIL signaling provide a biologically reliable framework for linking visceral obesity to IgAN progression, it is important to emphasize that most of the available studies use BMI rather than direct measurements of visceral obesity. Therefore, the link between VAT and IgAN remains hypothetical and has not yet been clinically proven. Future prospective studies, particularly those involving imaging-based VAT quantification and standardized metabolic assessments, are needed to verify whether visceral obesity exerts a unique and independent effect beyond BMI in IgAN.

### 4.3. Body Mass Index

A key limitation of these findings is the reliance of most studies on BMI as a substitute for obesity. Although BMI is widely used, it does not differentiate fat ranges or reflect visceral fat inflammatory load (VAT). This limitation explains why BMI-based evidence dominates previous studies: more precise markers—including waist circumference, weight-corrected waist index (STIs), and imaging-based VAT measurements—provide stronger links to IgAN progression and should be prioritized in future studies.

There are discrepancies regarding BMI as a marker of IgAN progression. BMI does not reflect the VAT burden and waist circumference is more accurate [22,30]. Underweight (BMI < 18.5 kg/m^2^) increases the risk of renal failure, which suggests a U-shaped relationship [54,55]. Some studies have not shown an effect of BMI on eGFR or proteinuria unless it is accompanied by hypertension or MetS [11]. The BMC Nephrology study from 2018 linked BMI to interstitial fibrosis (overweight OR 2.28; obesity OR 3.43) [20]. Only one study analyzed the risk of BMI and fibrosis [19].

An important observation is that BMI ≥ 25 kg/m^2^ combined with unfavorable histopathological features (mesangial hypercellularity, segmental sclerosis, IF/TA) significantly delays proteinuria remission, indicating synergistic enhancement of injury. To date, only one study has addressed this issue, indicating the need for further research.

Obesity as a chronic, multisystem disease remains insufficiently recognized [35]. VAT mediators promote autoimmunity, hyperfiltration, oxidative stress and structural changes (glomerular enlargement, basal membrane thickening), worsening IgAN [9,25,31,43]. The current KDIGO 2024 and ERA 2022 guidelines recognize obesity as a risk factor for CKD but provide limited recommendations [36]. In turn, the European Society of Cardiology (ESC) 2024 guidelines emphasize the treatment of obesity, including pharmacotherapy (semaglutide, tirzepatide) and surgery [47]. A 2023 BMC Nephrology study identified weight-adjusted waist index as the best predictor of CKD and albuminuria [56].

While many mechanisms (VAT cytokines, insulin resistance, hypertension, dyslipidemia) are common to the etiology of CKD, IgAN is unique due to its immune-mediated mechanisms (Gd-IgA1, BAFF/APRIL, complement). Future comparative studies should clarify whether the synergy of obesity and MetS is stronger in IgAN than in other causes of CKD.

#### 4.3.1. Limitation

This review has a few limitations. First, there is heterogeneity in obesity measurements, with most studies based on BMI, which does not include VAT or inflammation [22,30]. Few studies have used waist indicators or advanced measurements such as bioimpedance [56].

Second, the evidence is largely cross-sectional or retrospective, lacking prospective or interventional designs. The heterogeneity of endpoints and therapies prevented stratification by treatment (ACE-i, SGLT2i, steroids/immunosuppressants) when assessing the effects of obesity. Subgroup analyses were rarely available.

Third, most of the data comes from Asian and European cohorts, which limits the possibility of generalization. Few studies stratified by gender, despite hormonal and metabolic differences that can affect results.

Fourth, the conflicting BMI results highlight the need for standardization of definitions and multimodal assessment of obesity [20].

Finally, while GLP-1 receptor agonists, dual GLP-1/GIP agonists, and bariatric interventions are promising, direct evidence of IgAN is rare and requires clinical trials [47].

#### 4.3.2. Clinical Implications and Future Directions

Obesity is a modifiable risk factor and should be routinely evaluated in the IgAN patient group. Based on the results of most studies, patients with IgAN should undergo treatment for obesity in the form of lifestyle interventions, pharmacotherapy for underlying conditions, or bariatric surgery for very high-risk cases. Systematic treatment of obesity allows for proactive control of renal and cardiovascular risk in IgAN.

Future research should prioritize longitudinal, multiethnic cohorts with direct assessment of visceral adiposity (VAT) to better delineate its role in IgAN progression beyond BMI-based classifications.

## Figures and Tables

**Table 1:** Effect of BMI on IgAN progression in cohort and meta-analytical studies. Results indicate adverse kidney outcomes with elevated BMI, including delayed proteinuria remission, interstitial fibrosis, and ESRD risk, although some studies indicate inconsistent or U-shaped.

Reference	Type of Study	Conclusion
Ouyang Y, et al. [12]	Cohort	Underweight increases the risk of kidney failure by 17.3% compared to 9.5% in obese people
Wang Q i wsp. [13]	Meta-Analyze	A high BMI increases the risk of kidney side effects (OR 2.43)
Kataoka H, et al. [17]	Cohort	BMI ≥ 25 kg/m^2^: independent predictor of creatinine growth (OR 7.4)
Shimamoto M, et al. [18]	Cohort	Obesity delays proteinuria remission by >5 years
Wu C, et al. [19]	Cohort	Overweight/obesity increases the risk of interstitial fibrosis (OR 2.28–3.43)
Berthoux F, et al. [20]	Cohort	High BMI: worse long-term outcomes
Hong YA, et al. [21]	Cohort	Obesity independently associated with severe mesangial matrix expansion
Wang S, et al. [22]	Cohort	High BMI: 6.01 mL/min/1.73 m^2^ lower eGFR
Ariyasu Y, et al. [23]	Cohort	Obesity contributes to the atrophy of the ear canals
Nagasawa Y, et al. [37]	Cohort	Uric acid in women increases the risk of disease progression (OR 1.33)
Liu M, i wsp. [38]	Meta-Analyze	Hyperuricemia + Vascular Damage: Synergistic Effects on Progression

**Table 2:** Synergistic effect of BMI and additional risk factors (hypertension, hyperuricemia, hypertriglyceridemia, histopathological abnormalities) on IgAN progression. The data illustrate the multiplied risk when obesity coexists with other metabolic or pathological factors.

Combination	Quantitative Data	Source
BMI + mesangial matrix expansion	Obesity independently associated (*p* = 0.020)	[21]
BMI + interstitial fibrosis	OR 2.28 (overweight) OR 3.43 (obesity)	[19]
BMI + metabolic factors	Remission of proteinuria delayed >5 years	[18]
BMI + histological parameters	26.0 MATRIX for BMI ≥ 25 + M1 + Max GA ≥ 42,900 μm^2^	[17]
BMI + Global Optical Score	WMD 1.22 (95% CI 0.70–1.74) higher in obese subjects	[13]
BMI + hypertension/proteinuria	Worse event-free survival (*p* < 0.0001)	[20]
BMI + GBM thickness	Significantly increased in obese subjects (*p* < 0.001)	[39]
BMI + hyperuricemia	64.4% vs. 37% and freezes compared to normal	[22]
BMI + hypertriglyceridemia	71.3% vs. 32.5% and freezes compared to normal	[22]
BMI + atrophy of the combat ducts	Obesity contributes to the decline of kidney function	[23]
BMI + progression of CKD	79.5% vs. 44.7% progression in obesity vs. normal	[40]
BMI + TyG-BMI	OR 8.39 (95% CI 1.66–42.39) for LVH	[41]

TyG—BMI—Triglycerides—Glucose Body Weight/Index.

**Table 3:** Comparison of clinical parameters in patients with IgAN with and without MetS. The data indicate significant differences in BMI, proteinuria, eGFR, hypertension and dyslipidemia, which confirms the role of MetS as an accelerator of IgAN progression.

Parameter	MetS (+)	MetS (−)	Difference	Source
Age (years)	38–42	32–35	+5–7 years	[10,11]
BMI (kg/m^2^)	26–30	22–24	+15–25%	[10,11]
eGFR (mL/min/1.73 m^2^)	70–80	85–95	−15–20%	[10,11]
Hypertension (%)	70–80%	30–40%	+40%	[10,11]
Proteinuria (g/day)	1.5–2.0	0.8–1.0	+50–100%	[10,11]
Renal survival	Worse	Better	HR 2.0–3.0 (ESRD risk)	[10,11,42]
Cardiovascular events	23/65	15/60	+8 events	[10]
Number of metabolic components	Higher	Dolny	Increased risk of events	[12]
Dyslipidemia (%)	71.3%	32.5%	+38.8%	[22]
Diabetes (%)	Greater prevalence	Lower prevalence	Increased risk of events	[10]
Albuminuria	Higher	Lower	Increased	[10]

## Supplementary Materials

The following supporting information can be downloaded at: https://www.mdpi.com/article/10.3390/nu17203307/s1.
