# Supplementary material for: Visceral Obesity and Metabolic Dysfunction in IgA Nephropathy: Nutritional and Metabolic Perspectives on Disease Progression
Source: Nutrients. 2025 Oct 21;17(20):3307. doi: 10.3390/nu17203307 (PMC12567062; doi:10.3390/nu17203307)
Supplement: Supplementary file 1 [file nutrients-17-03307-s001.zip › nutrients-3851986-supplementary.pdf]

Table S1. Description of the Included Studies

	**Table 1. Description of the Included Studies**				
**No.**	**Article**	**Type of Study**	**Description of the Experiment and Study Groups/Description of the Study**	**Method of Analysis**	**Conclusion**
**1**	**Wyatt RJ, Julian BA. IgA nephropathy. N Engl J Med. 2013;368(25):2402-2414.**	**Review**	**Comprehensive review of IgA nephropathy (IgAN) epidemiology, pathogenesis, and clinical features. Discusses the four-hit model and genetic/environmental factors.**	**Narrative synthesis of literature on IgAN pathogenesis and risk factors.**	**IgAN is the most common primary glomerulopathy, with a complex pathogenesis involving genetic and environmental factors, including potential links to obesity.**
**2**	**Kovesdy CP, et al. Physiol Int. 2017;104(1):1-14.**	**Review**	**Examines the hidden consequences of obesity on kidney disease, including IgAN, focusing on mechanisms like inflammation and glomerular**	**Narrative synthesis of studies on obesity and CKD mechanisms.**	**Obesity is a significant risk factor for CKD progression, with potential implications for IgAN management.**

			**hyperfiltration.**		
**3**	**Suzuki H, et al. J Am Soc Nephrol. 2011;22(10):1795-1803.**	**Review**	**Details the pathophysiology of IgAN, focusing on the four-hit model: Gd-IgA1 production, autoantibody formation, immune complex deposition, and renal damage.**	**Narrative synthesis of molecular and immunological studies.**	**The four-hit model provides a framework for understanding IgAN progression, with potential overlap with obesity-related mechanisms.**
**4**	**Zhang H, et al. Front Med (Lausanne). 2023;10:1128393.**	**Review**	**Explores intrarenal inflammation in IgAN, focusing on molecular pathways (NF-κB, NOS) and cytokines (IL-6, TNF-α).**	**Narrative synthesis of inflammatory pathways in IgAN.**	**Inflammation is a key driver of IgAN progression, with potential exacerbation by obesity-related cytokines.**
**5**	**Ma X, et al. Ren Fail. 2025;47(1):2465810.**	**Review**	**Investigates the role of endothelin-1 (ET-1) in kidney diseases, including IgAN, and its**	**Narrative synthesis of ET-1 studies in renal pathology.**	**ET-1 exacerbates IgAN progression, potentially worsened by obesity-induced**

			**contribution to vasoconstriction and fibrosis.**		**endothelial dysfunction.**
**6**	**Kawai T, et al. Am J Physiol Cell Physiol. 2021;320(3):C375-C391.**	**Review**	**Analyzes adipose tissue inflammation and its role in obesity-related metabolic dysfunction, including renal effects.**	**Narrative synthesis of adipose tissue studies.**	**Visceral adipose tissue (VAT) drives inflammation, contributing to kidney damage in IgAN.**
**7**	**Tucker PS, et al. Oxid Med Cell Longev. 2015;2015:806358.**	**Review**	**Describes the relationship between oxidative stress, inflammation, and CKD, including IgAN, with a focus on obesity.**	**Narrative synthesis of oxidative stress studies.**	**Obesity-induced oxidative stress accelerates CKD progression, including in IgAN.**
**8**	**Zhu Y, et al. Nephrol Dial Transplant. 2024;39(10):1624-1641.**	**Experimental**	**Investigates the gut microbiome's role in Gd-IgA1 production in IgAN via the TLR4 signaling pathway, with obesity as a modulator.**	**16S rRNA sequencing, TLR4 signaling analysis, in vitro/in vivo models.**	**Obesity-related gut dysbiosis enhances Gd-IgA1 production, worsening IgAN.**

**9**	**Hall JE, et al. Circ Res. 2015;116(6):991-1006.**	**Review**	**Examines obesity-induced hypertension and its renal mechanisms, including implications for IgAN.**	**Narrative synthesis of hypertension and renal studies.**	**Obesity-related hypertension contributes to IgAN progression via glomerular hyperfiltration.**
**10**	**Bansal A, Chonchol M. Kidney Int. 2025;doi:10.1016/j.kint.2025.01.044.**	**Review**	**Introduces metabolic dysfunction-associated kidney disease (MDAKD) and its relevance to IgAN.**	**Narrative synthesis of MDAKD studies.**	**MDAKD links visceral obesity and metabolic dysfunction to IgAN progression.**
**11**	**Li X, et al. BMC Nephrol. 2023;24(1):266.**	**Cross-sectional**	**Analyzes the weight-adjusted waist index (WWI) as a predictor of CKD and albuminuria in 12,000 participants.**	**Logistic regression, odds ratio (OR) calculation, biomarker analysis.**	**WWI is a better predictor of CKD risk than BMI, relevant for IgAN management.**
**12**	**Nakayamada S, Tanaka Y. Inflamm Regen. 2016;36:6.**	**Review**	**Discusses BAFF and APRIL in autoimmune diseases, including their role in IgAN and obesity.**	**Narrative synthesis of BAFF/APRIL studies.**	**BAFF and APRIL contribute to IgAN autoimmunization, potentially exacerbated by obesity.**

13	Kim DH, Do MS. Exp Mol Med. 2015;47(1):e129.	Experimental	BAFF knockout in mice reduces systemic inflammation and obesity-related insulin resistance.	Mouse models, biochemical assays, cytokine analysis.	BAFF depletion ameliorates obesity-related inflammation, relevant to IgAN.
14	Kim B, Hyun CK. Int J Mol Sci. 2020;21(14):5121.	Experimental	BAFF depletion enhances thermogenesis in adipose tissue, reducing insulin resistance.	Mouse models, thermogenesis assays, cytokine analysis.	BAFF links obesity and IgAN via inflammation and insulin resistance.
15	Cheung CK, et al. Front Nephrol. 2023;3:1346769.	Review	Explores the role of BAFF and APRIL in IgAN pathogenesis and potential therapeutic targets.	Narrative synthesis of BAFF/APRIL studies in IgAN.	BAFF and APRIL are key players in IgAN, with potential obesity-related exacerbation.
16	Forte N, et al. Int J Mol Sci. 2020;21(5):1554.	Review	Examines the microbiota-gut-brain axis and its regulation by obesity-related mediators.	Narrative synthesis of microbiome studies.	Obesity-induced gut dysbiosis contributes to IgAN via the gut-kidney axis.
17	Francisco V, et al. Front Physiol. 2018;9:640.	Review	Analyzes leptin's role in obesity and immune system	Narrative synthesis of leptin studies.	Leptin enhances B-cell activity, worsening IgAN in

			**modulation, including in IgAN.**		**obese patients.**
**18**	**Kwiat VR, et al. Front Physiol. 2022;13:887702.**	**Review**	**Discusses autoimmunity as a consequence of obesity and systemic inflammation.**	**Narrative synthesis of autoimmunity studies.**	**Obesity amplifies autoimmunization, accelerating IgAN progression.**
**19**	**Puthumana J, et al. J Clin Invest. 2021;131(3):e139927.**	**Cohort**	**Investigates inflammatory biomarkers (hsCRP, YKL-40) in CKD progression, including IgAN.**	**Biomarker assays, regression models, survival analysis.**	**Inflammatory markers are elevated in IgAN with obesity, correlating with worse outcomes.**
**20**	**Camilla R, et al. Clin J Am Soc Nephrol. 2011;6(8):1903-11.**	**Cohort**	**Examines oxidative stress and Gd-IgA1 as progression markers in 200 IgAN patients.**	**Biomarker assays, survival analysis, correlation models.**	**Oxidative stress, exacerbated by obesity, predicts IgAN progression.**
**21**	**Barratt J, et al. Kidney Int. 2023;104(2):254-264.**	**Review**	**Discusses the lectin pathway in IgAN and its therapeutic implications.**	**Narrative synthesis of complement pathway studies.**	**Complement activation, worsened by obesity, drives IgAN progression.**
**22**	**Shim K, et al. World J Diabetes. 2020;11(1):1-12.**	**Review**	**Analyzes complement activation in obesity and**	**Narrative synthesis of**	**Obesity enhances complement activation,**

			type 2 diabetes, with renal implications.	complement studies.	contributing to IgAN damage.
23	Nigro E, et al. Biomed Res Int. 2014;2014:658913.	Review	Examines adiponectin's role in obesity and obesity-related diseases, including CKD.	Narrative synthesis of adiponectin studies.	Low adiponectin in obesity worsens IgAN progression.
24	Ahmed N, et al. Nat Rev Nephrol. 2025;21:417–434.	Review	Discusses adipose tissue-derived signals and their impact on kidney function.	Narrative synthesis of adipose tissue studies.	VAT-derived signals accelerate IgAN progression via inflammation.
25	Thomsen SB, et al. PLoS One. 2015;10(7):e0133672.	Cohort	Analyzes YKL-40 and its association with obesity and dyslipidemia in 1,500 high-risk patients.	Biomarker assays, regression models, correlation analysis.	YKL-40 is elevated in obese IgAN patients, correlating with progression.
26	Cebeci E, et al. Exp Clin Endocrinol Diabetes. 2019;127(4):189-194.	Cohort	Investigates resistin levels in type 2 diabetes and CKD, including IgAN.	Biomarker assays, correlation analysis, regression models.	Inflammation, not obesity alone, drives resistin levels in IgAN.

**27**	**Arrizabalaga P, et al. Med Clin (Barc). 2001;117(9):321-325.**	**Cohort**	**Examines ICAM-1 expression in 50 IgAN patients and its correlation with renal damage.**	**Immunohistochemistry, statistical analysis, correlation models.**	**ICAM-1 is elevated in IgAN with obesity, linked to tubulointerstitial damage.**
**28**	**Bae EH, et al. Nutrients. 2021;14(1):154.**	**Cohort**	**Nationwide study of 500,000 diabetic patients, assessing underweight and weight change effects on ESRD.**	**Cox regression, hazard ratio (HR) calculation, survival analysis.**	**Underweight increases ESRD risk, relevant to IgAN management.**
**29**	**Engelsen SJ, et al. Cardiovasc Diabetol. 2012;11:25.**	**Cross-sectional**	**Analyzes hsCRP in 300 centrally obese patients with MetS.**	**Biomarker assays, regression models, correlation analysis.**	**hsCRP is elevated in MetS, correlating with IgAN progression.**
**30**	**Nagaraju SP, et al. Saudi J Kidney Dis Transpl. 2018;29(2):318-325.**	**Cohort**	**Examines BMI's impact on IgAN progression in 150 Indian patients.**	**Regression analysis, survival models, correlation analysis.**	**BMI has no independent effect on IgAN progression in this population.**
**31**	**Sági B, et al. Biomedicines. 2024;12(6):1250.**	**Cohort**	**Compares renal and cardiovascular outcomes in 125 IgAN patients with/without MetS.**	**Survival analysis, logistic regression, event rate analysis.**	**MetS increases endpoint events in IgAN, worsening prognosis.**

**32**	**Ma H, et al. Sci Rep. 2024;14(1):30996.**	**Cohort**	**Analyzes metabolic component count and IgAN prognosis in 200 patients.**	**Cox regression, HR calculation, survival analysis.**	**Higher MetS components predict worse IgAN outcomes.**
**33**	**Ouyang Y, et al. PLoS One. 2016;11(9):e0162044.**	**Cohort**	**Investigates underweight as a risk factor for renal deterioration in 250 IgAN patients.**	**Survival analysis, OR calculation, regression models.**	**Underweight increases renal deterioration risk in IgAN.**
**34**	**Wang Q, et al. Int Urol Nephrol. 2022;54(5):1067-1078.**	**Meta-analysis**	**Systematic review and meta-analysis of BMI's impact on IgAN prognosis (10 studies, 2,000 patients).**	**Meta-reggression, OR calculation, heterogeneity analysis.**	**High BMI increases adverse renal outcomes in IgAN (OR 2.43).**
**35**	**Kataoka H, et al. Clin Exp Nephrol. 2012;16(5):706-712.**	**Cohort**	**Examines BMI and histopathological parameters in 100 IgAN patients.**	**Regression analysis, survival models, histopathological scoring.**	**Obesity delays proteinuria remission in IgAN.**
**36**	**Shimamoto M, et al. J Clin Lab Anal. 2015;29(5):353-360.**	**Cohort**	**Analyzes BMI's impact on IgAN progression in 150 Japanese patients.**	**Regression analysis, OR calculation, survival models.**	**High BMI is linked to worse IgAN outcomes.**

**37**	**Wu C, et al. BMC Nephrol. 2018;19(1):381.**	**Cohort**	**Investigates BMI and interstitial fibrosis in 200 IgAN patients.**	**Logistic regression, OR calculation, histopathological analysis.**	**Obesity increases interstitial fibrosis risk in IgAN.**
**38**	**Berthoux F, et al. Nephrol Dial Transplant. 2013;28 Suppl 4:iv160-166.**	**Cohort**	**Examines obesity as a risk factor in 300 IgAN patients.**	**Survival analysis, OR calculation, regression models.**	**Obesity is an independent predictor of IgAN progression.**
**39**	**Hong YA, et al. J Clin Med. 2020;9(9):2824.**	**Cohort**	**Analyzes obesity's impact on clinicopathologic parameters in 250 IgAN patients.**	**Regression analysis, correlation models, histopathological scoring.**	**Obesity worsens IgAN severity and progression.**
**40**	**Wang S, et al. Front Endocrinol (Lausanne). 2023;14:1094534.**	**Cohort**	**Examines obesity and ESRD risk in 200 IgAN patients.**	**Cox regression, HR calculation, survival analysis.**	**Obesity is linked to higher ESRD risk in IgAN.**
**41**	**Ariyasu Y, et al. Clin Exp Nephrol. 2024;28(11):1155-1167.**	**Cohort**	**Analyzes obesity's impact on IgAN prognosis by sex in 300 patients.**	**Regression analysis, survival models, sex-stratified analysis.**	**Obesity affects IgAN progression more in females.**
**42**	**Nagasawa Y, et al. PLoS One. 2016;11(8):e0160828.**	**Cohort**	**Examines uric acid's impact on IgAN progression in 200 patients,**	**Survival analysis, OR calculation, regression models.**	**Uric acid predicts IgAN progression in females.**

			stratified by sex.		
43	Versini M, et al. Autoimmun Rev. 2014;13(9):981-1000.	Review	Discusses obesity's role in autoimmune diseases, including IgAN.	Narrative synthesis of autoimmune studies.	Obesity amplifies autoimmune responses in IgAN.
44	Liu M, et al. Medicine (Baltimore). 2022;101(46):e31824.	Meta-analysis	Systematic review of obesity's impact on IgAN in Asians (8 studies, 1,500 patients).	Meta-regression, OR calculation, heterogeneity analysis.	Weight gain promotes IgAN progression in Asians.
45	Tanaka M, et al. Nephron Clin Pract. 2009;112(2):c71-78.	Cohort	Compares ultrastructural changes in obese vs. non-obese IgAN patients (50 patients).	Electron microscopy, statistical analysis, histopathological scoring.	Obesity is linked to severe mesangial matrix expansion.
46	Othman M, et al. Nephron Clin Pract. 2009;113(1):c16-23.	Cohort	Examines obesity's impact on non-diabetic CKD, including IgAN, in 200 patients.	Survival analysis, regression models, biomarker analysis.	Obesity accelerates CKD progression, including IgAN.
47	Jv M, et al. Eur J Med Res. 2024;29(1):627.	Cohort	Analyzes TyG-BMI index and left ventricular hypertrophy	Regression analysis, OR calculation, biomarker analysis.	TyG-BMI predicts adverse outcomes in IgAN.

			**in 150 IgAN patients.**		
**48**	**Qin A, et al. J Clin Med. 2022;11(18):5386.**	**Cohort**	**Examines TyG index and renal survival in 200 IgAN patients.**	**Survival analysis, HR calculation, regression models.**	**TyG index predicts worse renal survival in IgAN.**
**49**	**Yoshida Y, et al. Intern Med. 2025;doi:10.2169/internalmedicine.4613-24.**	**Cohort**	**Analyzes obesity, visceral fat, and dyslipidemia in CKD risk (300 patients).**	**Regression analysis, OR calculation, biomarker analysis.**	**Visceral fat increases CKD risk, relevant to IgAN.**
**50**	**Garofalo C, et al. Kidney Int. 2017;91(5):1224-1235.**	**Meta-analysis**	**Systematic review of obesity and CKD onset in the general population (15 studies, 10,000 patients).**	**Meta-regression, OR calculation, heterogeneity analysis.**	**Obesity predicts CKD onset, with implications for IgAN.**
**51**	**Kotsis V, et al. Nutrients. 2021;13(12):4482.**	**Review**	**Discusses obesity's impact on kidney diseases, including IgAN.**	**Narrative synthesis of obesity and CKD studies.**	**Obesity worsens kidney disease outcomes, including IgAN.**
**52**	**D'Agati VD, et al. Nat Rev Nephrol. 2016;12(8):453-471.**	**Review**	**Examines obesity-related glomerulopathy and its**	**Narrative synthesis of glomerulopathy studies.**	**Obesity-related glomerulopathy exacerbates IgAN**

			**overlap with IgAN.**		**progression.**
**53**	**Yang S, et al. Kidney Blood Press Res. 2020;45(4):510-522.**	**Review**	**Discusses latent changes in obesity-related glomerulopathy requiring attention in IgAN.**	**Narrative synthesis of glomerulopathy studies.**	**Obesity-related glomerulopathy worsens IgAN outcomes.**
**54**	**Zhang J, et al. Clin Exp Nephrol. 2021;25(8):865-874.**	**Cohort**	**Compares obesity-related glomerulopathy and IgAN in 100 patients.**	**Regression analysis, survival models, histopathological scoring.**	**Overlap of obesity-related glomerulopathy and IgAN worsens prognosis.**
